# Correlation of COVID-19 Pandemic with Healthcare System Response and Prevention Measures in Saudi Arabia

**DOI:** 10.3390/ijerph17186666

**Published:** 2020-09-13

**Authors:** Heba M. Adly, Imad A. AlJahdali, Mohammed A. Garout, Abdullah A. Khafagy, Abdulla A. Saati, Saleh A. K. Saleh

**Affiliations:** 1 Community Medicine and Pilgrims Health Department, Faculty of Medicine, Umm Al-Qura University, Mecca 24381, Saudi Arabia; hebamadly@hotmail.com (H.M.A.); iajahdali@uqu.edu.sa (I.A.A.); magarout@uqu.edu.sa (M.A.G.); aakhafagy@uqu.edu.sa (A.A.K.); aaasaati@uqu.edu.sa (A.A.S.); 2 Biochemistry Department, Faculty of Medicine, Umm Al-Qura University, Mecca 24381, Saudi Arabia; 3 Oncology Diagnostic Unit, Faculty of Medicine, Ain Shams University, Abbassia, Cairo 11566, Egypt

**Keywords:** COVID-19, pandemic, prevention, healthcare, Saudi Arabia, organizational measures, environmental measures, infection, coronavirus, SARS-CoV-2, COVID-19, protection, protocol, transmission

## Abstract

Background: The Saudi government has taken the decision to prevent the entrance of about 2.5 million international pilgrims seeking to perform hajj in order to protect the world from a catastrophic widespread of disease. Moreover, health systems in Saudi Arabia are offering free testing for residents whether Saudi and non-Saudi. Objective: This study aimed to evaluate the spread of COVID-19 associated with preventive measures taken in Saudi Arabia and to develop a detailed COVID-19 prevention strategy as a framework for the Saudi Arabia community. Methodology: Population size and age distributions among the country of Saudi Arabia were taken from the 2020 World Population Prospects. Contact patterns were measured using the Saudi Arabia Ministry of Health Statistical Annual Report. Conclusions: Our study demonstrates that performing screening tests as early as possible to facilitate the rapid detection of infected cases, fast treatment, and instant isolation for suspected cases is the most definitive rejoinder for public health. Moreover, our study revealed the significance of performing preventive measures in reducing infection and death rates around Saudi Arabia by 27%, while in other countries, it reduced the death rate ranging from 10–73%. This study provides an achievable strategy for prevention and early detection of COVID-19 spread.

## 1. Introduction

An extremely infectious new coronavirus has arisen as a COVID-19 outbreak and refers to the SARS CoV-2 virus. The COVID-19 on going pandemic has propagated rapidly and has sparked a universal health crisis [1]. As COVID-19 spread through human breathing droplet infections and direct contact with diseased individuals have been studied widely—as indicated on 22 July 2020—the COVID-19 pandemic has resulted in about 14,747,822 cases worldwide including 610,791 deaths and 8,803,885 recovered cases [2]. Currently, infected cases are at 5,333,146 from which 5,273,445 (99%) are mild conditions while (1%) 59,701 are serious or critical cases [2]. Saudi Arabia has recorded 250,920 confirmed cases [3].

### 1.1. Demographics and COVID-19 Cases

Some studies have shown that higher profit nations have the eldest residents, unlike lower income countries that have a slightly number of residents who are above 65 and therefore within the stage interval now detected to be at remarkably high risk of mortality from COVID-19 [4]. The family unit is a vital perspective for COVID-19 transmission. The normal size of families with an occupant over the age of 65 years is considerably higher in countries with lower income associated with moderate- and high-income populations, increasing the capacity for spread mostly to this susceptible age-group [5]. Communication models between age-groups also vary by country; in high-income sites, contact models are likely to decrease suddenly with age. This effect is more reasonable in middle-income locations and vanishes in low-income settings, demonstrating that elderly persons in these locales (LICs and MICs) sustain higher contact rates with a wide variety of age-groups compared to elderly persons in high-income countries (HICs). These contact forms affect the predicted COVID-19 infection outbreak rate across age-groups with higher occurrence rates in the elderly projected in low-income locations associated with high-income locations and middle-income screening middle designs [6].

Saudi Arabia is distributed into 13 provinces with a total population of 34,813,871 [7]. The 2019 population density in Saudi Arabia was 16 people per km^2^ estimated on an overall land-living area of 2,149,690 km^2^. The Saudi elderly (65+ years) represents 81.3%. Of this ratio, 40% are male and 41.9% are female. The percentage of the elderly (65+ years) is the lowest in the Northern Borders Region (1.07%), however, are at the top of the list in Makkah (29.4%). The relative number of Saudi elderly (65+ years) tops the list in Makkah (24.5%), although achieved the lowest in Northern Borders Region (1.3%). Furthermore, the percentage of the male Saudi elderly (65+ years) tops the list in Makkah (24.4%), but was the lowest in the Northern Borders Region (1.2%) out of the total Saudi population [7,8].

### 1.2. Healthcare Availability and COVID-19 Scattering

Hospital bed capacity is greatly associated with the income level of countries. Low income countries have the smallest number of hospital beds per 1000 population (1.24 beds per 1000 population as average) while high income countries have the highest (4.82 beds per 1000 population as average). Lower and upper middle-income countries (LMIC/UMICs) are sandwiched between these two boundaries (2.08 and 3.41 beds per 1000 population on average, correspondingly) [9]. The percentage of hospital beds that are in intensive care units (ICU) is lowest in LICs (1.63 on average) and highest in HICs (3.57), with LMICs and UMICs falling in-between (2.38 and 3.32, respectively) [10].

In Saudi Arabia, the Ministry of Health (MoH) monitors all healthcare activities and services inside the country. MoH has performed a leading role in delivering healthcare services including preventive, curative, and rehabilitation activities in Saudi Arabia. By launching Public Private Participation (PPP) healthcare models, the government is working in the direction of revealing value in the health system and fast-tracking healthcare transformation with the intention to increase private sector involvement in total healthcare spending to 35% by 2020 [11]. In 2019, the MoH provided 58.3% of the total hospitals and 59.1% of the total bed supply [11]. Moreover, there are quasi-government healthcare facilities that are hospitals and health centers controlled by the MoH and primarily accommodates workers of these government associations. Some of the quasi-government facilities consist of the National Guard, Ministry of Defense and Aviation, Ministry of Interior, Royal Commission. Finally, private sector services are reclaimed by emigrants when they do not have access to public services, and at times, Saudi nationals also go to private facilities to avoid the waiting time at public facilities and gain from the higher quality of care. The private sector operates approximately 32.2% of hospitals, carrying 24.6% of the overall bed supply [12].

### 1.3. COVID-19 Epidemiology

The epidemiological records correlated to the SARS-CoV-2 (COVID-19) disease is continually progressing and is revised daily in the COVID-19 condition report by the World Health Organization [13]. Referring to the original study in Wuhan, with a small sample of 99 individuals, the contagion was more likely to affect old age males with comorbidities, which encouraged the appearance of characteristic occurrences of serious or even fatal respiratory pathologies as acute respiratory distress syndrome [14]. The figures have been long-established by the main epidemiological project shown by the Chinese Center for Disease Control and Prevention, which found that the mortality rate increased from 0.2% in patients aged between 10 and 39 years to 14.8% in those over 80, and the death risk was greater between men (2.8%) than between women (1.7%). An Additional vital issue has been found in the synchronized existence of pre-existing diseases, particularly cardiovascular and metabolic pathologies such as diabetes, chronic respiratory failure, and hypertension. Nevertheless, amongst those who were in wonderful health at the time of infection, the mortality rate was 0.9%. Moreover, it has been revealed that 80.9% of infections have no symptoms or mild ones and 13.8% are severe, whereas 4.7% of individuals infected developed critical pathological symptoms with symptoms such as respiratory failure, septic shock, or multi-organ failure [15].

The basic reproduction number (R0), defined as the mean figure of secondary cases created by primary cases as people are generally vulnerable to infection, defines the overall number of people expected to be infected, or more accurately, the area below the outbreak curve. Designed for an epidemic to take on, the value of R0 must be larger than the unity in value [16]. In the influenza A H1N1 pandemic in 2009, for most infected people, the epidemiological amounts were rapid with a day or two to infectiousness and a few days of peak infectiousness to others [17]. In comparison, for COVID-19, the sequential period is projected to be 4.4 to 4.7 days, which is more comparable to SARS [18]. The top estimations indicate a case fatality rate (CFR) for COVID-19 of nearly 0.3 to 1%, which is higher than the order of 0·1% CFR for a moderate influenza A season [19].

Studies have shown that the epidemic in any specified country will originally spread more gradually than is characteristic for a new influenza A strain. COVID-19 had a double up time in China of about 4–5 days in the early phases [20]. The COVID-19 epidemic might be more sketched out than seasonal influenza A, which is of relevance for its possible economic effects. As the effect of seasons on the transmission of COVID-19 remains unidentified [21]; though, along with an R0 of 2–3, the hot months of summer in the Northern Hemisphere may possibly decrease transmission under the value of unity as for influenza A, which usually has an R0 of around 1.1–1.5 [22]. Strongly related to these factors and its epidemiological determining factor is the influence of various mitigation strategies of the COVID-19 epidemic [23].

Statistics published from China, South Korea, Italy, and Iran propose that the CFR increases abruptly with age and is sophisticated in patients with COVID-19 and primary comorbidities [24]. Social distancing for such groups might be the greatest operative method to decrease morbidity and attendant mortality. Throughout the Ebola virus disease outbreak in West Africa from 2014–2016, deaths from additional causes has increased for a saturated health-care system and deaths of health-care workers [25]. The USA counts statistics for COVID-19 epidemics through studying the mortality statistics time series by employing a time-shifting autoregressive state-space model. The preliminary spread differed significantly between states, with the highest R0 = 0.31 in New York State, which was R0 = 6.4. The change in original R0 was greatly associated with the highest daily death calculation amongst states, illustrating that the initial R0 predicts consequent questions in managing epidemics [26].

### 1.4. Prevention Measures and COVID-19

The government-commenced interference strategies may be comparatively short-term, but the severe and long-long-term economic and social outcomes are to be reduced. Though the 76-day over the city quarantine in Wuhan, composed with weeks of country wide emergency responses against the epidemic, have mostly measured the SARS-COV-2 in the country, it originated with massive economic significances that showed that local product shriveled by 6.8% in the 2020 first quarter [27]. Regrettably, the risk of a renaissance of the epidemic from smuggled cases persists at a high level with different control procedures [27]. Currently, a minimum of six countries including China, Germany, Iran, South Korea, Lebanon, and Saudi Arabia have undergone several resurgences of the epidemic after ending the lockdown [28].

Mass awareness is significant in assisting and maintaining government interferences [29]. Greater awareness must specifically focus on individuals with a lesser awareness such as elderly people and individuals of lower level of education [30]. The information from the United States shows that cultural minorities with socioeconomic difficulties are inclined to be at a high-risk level of acquiring COVID-19 [31]. Numerous considerations such as living in dense population areas, inadequate personal and environmental hygiene, and minimal healthcare affordability give rise to a higher risk of COVID-19 infection in these populations. Personal awareness of protective measures is consequently more vital in these inhabitants to prevent a wide-spreading epidemic [32].

Moreover, governments need to confirm their achievements to most of the crucial groups who have assumed that social distancing will have limited some communication. Social media is an active resource of statement to spread COVID-19 epidemic evidence and enhance mass awareness for epidemic prevention such as encouraging proper handwashing. Nevertheless, elderly inhabitants might be not familiar with the fast development of social media, and standard methods of mass media such as radio, television, mail, and telemarketing should be applied to reach these groups. On the other hand, social media can also circulate misinformation that the WHO has so-called an ‘infodemic’, which is noticeable with disproportionate untrue content, gossip, and misinformation that may counteract the profits of mass awareness and bring uncertainty and panic [33]. Studies have shown that a mixture of self-enforced approaches can be undertaken more readily than only isolated prevention. Social distancing and handwashing are useful strategies that are well agreed upon by most people, however, the significance of face mask use cannot be ignored. Although there is a continuing argument on whether it is necessary to wear face masks by non-medical persons, the effectiveness of face masks has been recognized in the preceding outbreaks of SARS and influenza [34], along with in clinical locations for COVID-19 [35]. Along with the prevention of the air spread of aerosols, face masks might also reduce the regular contacts between the hand and nose. A social study described that people touch their face 23 times every hour with no notice [36]. Additionally, an accruing body of modeling studies show a significant advantage from face mask use by the public [37]. Moreover, environmental procedures are designed to reduce the transmission risk of SARS-CoV-2 to persons through contact with infected persons, items, apparatus, or polluted environmental surfaces. Protective devices should be worn on every occasion that there is possibly a close interaction with a suspect case, particularly if the possibly infected individual does not wear a surgical mask that may reduce the dispersal of the virus in the environment. By implementing this prevention and protection measure endorsed in a place of work, it will potentially improve to defeat this COVID-19 pandemic [38]. Governments must consequently rapidly take accurate and inclusive measures to prevent the spread of the epidemic [39].

This study aimed to evaluate the spread of COVID-19 associated with preventive measures taken in Saudi Arabia and to assess the COVID-19 mortality rate related to pandemic control actions taken by healthcare systems in Saudi Arabia and to develop a detailed COVID-19 prevention protocol as a framework to the Saudi Arabian community.

## 2. Materials and Methods

### 2.1. Demography and Patterns of Contact across Saudi Arabia

Population size and age distributions among the country of Saudi Arabia were taken from the 2020 World Population Prospects, the 27th round of the official United Nations population estimations organized by the Population Division of the Department of Economic and Social Affairs of the United Nations Secretariat [40].

The patterns of contact among Saudi populations in different provinces were taken from several sources including the Colliers report and The Pulse: 8th edition, Kingdom of Saudi Arabia Healthcare overview 2018 [8]. Additional data were obtained from a survey of socioeconomic levels of different countries included in the socialmixR packages [41]. Finally, contact patterns were measured using the Saudi Arabia Ministry of Health Statistical Annual Report [42,43].

The number of cases included in the study were identified as persons with SARS-CoV-2 infection confirmed with reverse transcriptase–polymerase chain reaction (RT-PCR) and received standard isolation treatment. Moreover, suspected individuals, known as any person who had direct contact with a person infected with SARS-CoV-2, had to offer a throat swab sample to test for the presence of SARS-CoV-2. This test was performed when the person was detected by the Novel Saudi Arabia Ministry of Health software applications “Tabaoud” and “Tawakkalna”. In the case the RT-PCR results were positive for SARS-CoV-2, persons were obliged to stay in the hospital, if not, individuals were isolated at a hotel under the control and care of physicians and nurses.

### 2.2. Community Prevention Program

The prevention platform for COVID-19 has been performed in the Saudi Arabia community since January 15, 2020. This study reports on the most important framework of this prevention package and evaluates the outcomes correlated with employing these precise control measures. The study protocol was approved by the Ethics Review Board for Human Studies at the Faculty of Medicine, Umm Al Qura University. There was no need for informed consent because data collection was performed as part of a public health investigation of the COVID-19 pandemic from the Ministry of Health registry.

This study subsequently describes the Strengthening the Reporting of Observational Studies in Epidemiology (STROBE) reporting guideline for all confirmed COVID-19 cases, contacts, and individuals in Saudi Arabia who were considered to be at high risk and were registered at hospitals from 1February to 27 July 2020.

The study analyzed and searched the evidence-based guidelines for community protection issued by the Saudi Ministry of Health affected by the COVID-19 pandemic and compared the published guidelines with other countries.

### 2.3. Study Limitations

Our study had a few limitations. All reported cases were analyzed in consideration of the announced numbers by the Ministry of Health ignoring lost and unknown cases as well as the comparatively short duration of follow-up, the outcomes related with the interference measures depicted in this study should be evaluated with concern. Consequently, more forthcoming cohort studies about community control strategies should be performed in the future including more and larger cases. Thus, community control policies may need adjustments for effective application.

### 2.4. Statistical Analysis

Statistical analysis data are shown as mean ± standard deviation (SD) for the various studied parameters and their correlations. Group data analysis was compared by using one-way variance (ANOVA), and the differences between the means of two of the three studied groups were analyzed using an independent-sample t-test. The significance level was *p* < 0.05. SPSS software (SPSS Inc.; Chicago, IL, USA) was used for the statistical analyses.

## 3. Results and Discussion

### 3.1. Correlations between Prevention Measures and Number of Cases

The Saudi Arabian population is around 34,813,867 with a population density of 15.322 and a median age of 31.5 years, with 3.295 at 65 years of age and 1.84 at age 70. Cardiovascular death rate was 42% and diabetes prevalence was 17.7%. The percentage of male and female smokers was 25.4% and 1.8%, respectively. In Saudi Arabia, the hospital bed number per thousand is 2.7 with a Saudi life expectancy of 75.13 years old [44].

Saudi Arabia is considered as one of the most impacted countries by the coronavirus pandemic and is striving to reduce the coronavirus rate. The Saudi government has made the decision to prevent the entrance of about 2.5 million international pilgrims seeking to perform the hajj this year. Moreover, several health systems in Saudi Arabia are offering inhabitants—whether Saudi residents or non-Saudi residents—access to testing practices to reduce the outbreak [45]. On 27 July, 2020, the Saudi Arabia Ministry of Health declared that about three million COVID-19 tests had been conducted since the beginning of the pandemic, and the total number of coronavirus cases reached 262,772, of which 2378 were new cases. The rise in confirmed cases took place as the daily tests at all healthcare facilities in the kingdom increased from 1000 to 65,000 tests daily. The active cases remain at 44,369 including 2143 critical ones, while 225,696 cases had an outcome from which 222,936 (99%) recovered or discharged and 2760 (1%) were deaths.

**Figure 1:**
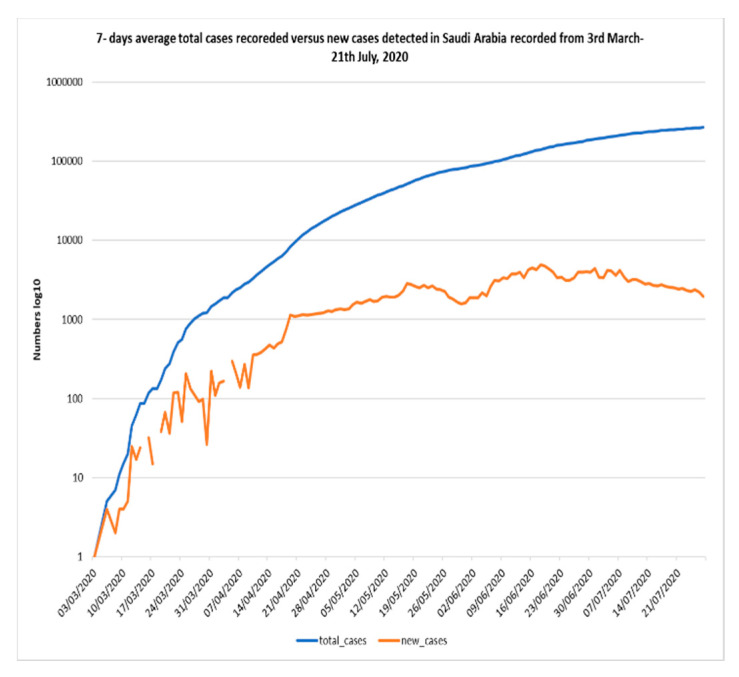
COVID-19 7-days average total cases versus new cases recorded in Saudi Arabia recorded from 3 March–21 July 2020 (*Using logarithmic scale based 10*).

**Figure 2:**
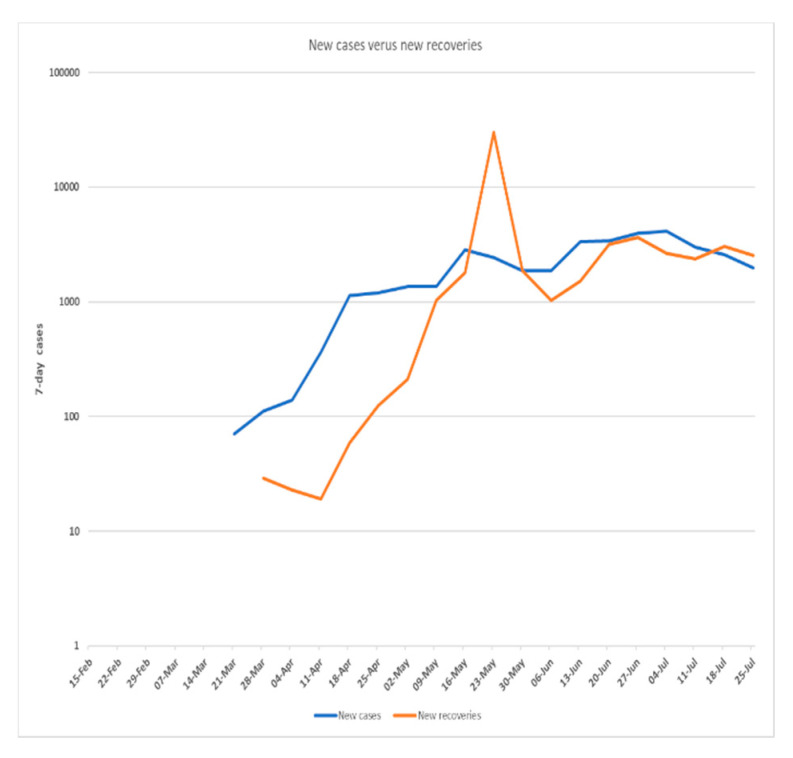
COVID-19 newly infected cases versus newly recovered cases in Saudi Arabia recorded from 15 February–21July 2020 (*Using logarithmic scale based 10*).

**Figure 3:**
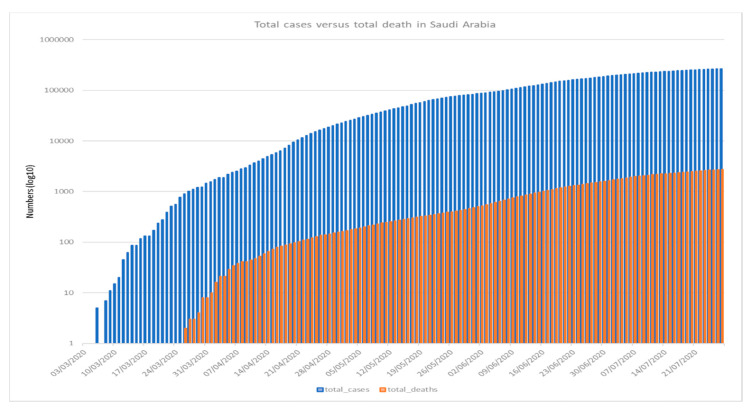
COVID-19 total recorded cases versus total death in Saudi Arabia recorded for COVID-19 from 3 March–21 July 2020 (*Using logarithmic scale based 10*).

Figure 1, Figure 2 and Figure 3 shows the 7-day detected total cases against new cases, the number of new cases versus recoveries, and the total cases recorded versus death numbers, respectively. These figures indicate that in Saudi Arabia, the current rate of transmission (R0) was estimated to be around 4 at the beginning of the pandemic in Saudi Arabia in March 2020. Then, the R0 decreased to be around 2–3 through March to June. At the beginning of July, the R0 was lowered to reach 1 around all Saudi cities. The global rate of transmission R0 ranged from 2.5–3.5 [46]. The decrease in R0 around Saudi cities is related to the country lockdown and government prevention measures.

**Figure 4:**
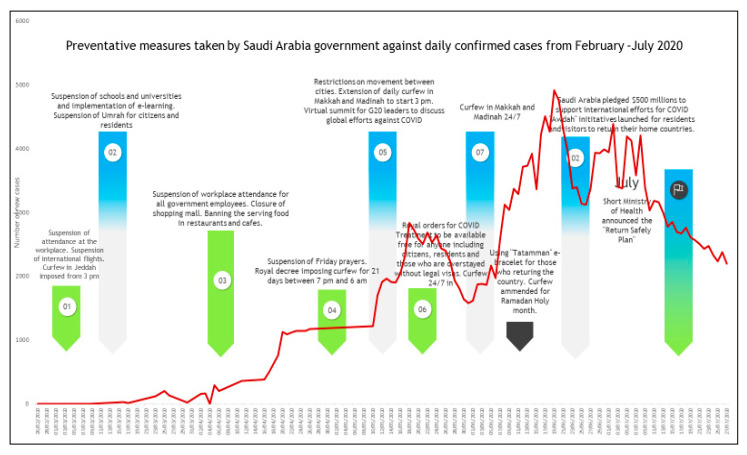
Timeline of preventive measures taken by the Saudi Arabian government compared with the daily COVID-19 confirmed new cases recorded from February 3–July 21 2020.

As reported in Figure 4, Saudi Arabia has launched several preventative measures starting on February 26. Despite these limitations, on March 2, Saudi Arabia reported its first COVID-19 confirmed case for a traveler returning from Iran via Bahrain without declaring their travel history to Iran. On March 4, Umrah was totally suspended and all pilgrimage travel plans were cancelled until further notice and the two holy mosques in Makkah and Madinah were set to completely close for cleaning and sterilization by March 5. On March 8, the Saudi government transferred schools and universities to distance learning and simulated classrooms. This was complemented by a travel prohibition to all countries and setting a compulsory quarantine for travelers who arrived from affected countries. On March 9, Saudi Arabia pledged 10 million U.S. dollars to the WHO to assist efforts to fight the pandemic. By March 12, all social and governmental crowds and social events were banned or postponed including the Saudi African and Arab-African summits. Consequently, all international and domestic air flights, sports events, and workplaces were postponed. In addition, the five daily prayers in all mosques around the country were forbidden and all Muslims in Saudi Arabia were requested by religious authorities to pray at homes for the very first time in the history of the Kingdom. This is considered as a significant stage considering that Saudi Arabian law is based on Islamic law and most Saudis carry out their prayers in mosques five times a day. Digital health was promptly stimulated and used for numerous essential services such as the “my health” application that allows people to seek medical support and obtain medical prescriptions without the necessity to visit medical centers physically. It was noticeable that most preventive measures taken by the government were implemented by March 15, meaning that only 300 cases from a 35 million population of about 0.03% per million. By 21 July, the confirmed new cases per million of Saudi population reached 7667,663 per million with the banning of international flights remaining in place and prohibitions of the Umrah and Hajj.

**Figure 5:**
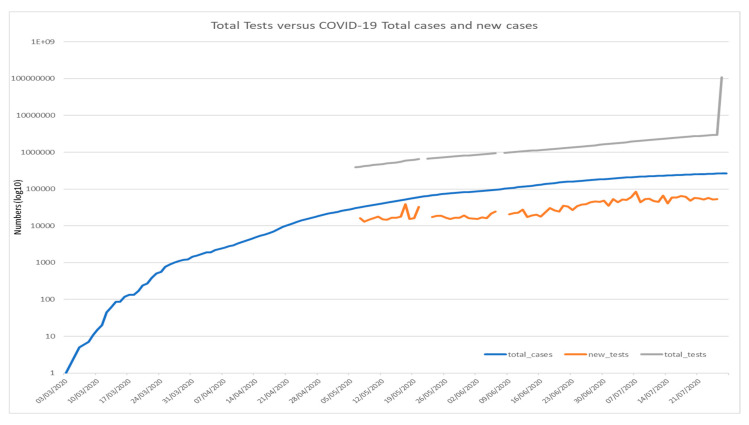
Cumulative tests performed against the daily COVID-19 confirmed new cases compared with cumulative cases recorded from March–July 2020 (*Using logarithmic scale based 10*).

**Figure 6:**
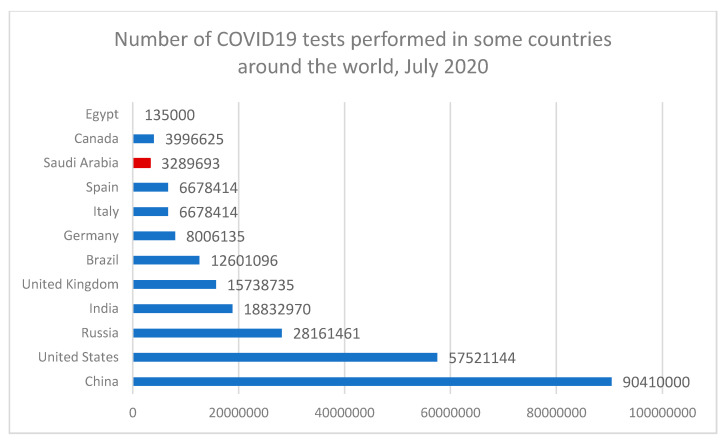
Number of COVID-19 tests performed in some countries around the world in July 2020.

The certainty of the actual total number of infected people around the world is still unconfirmed. The only way to know a country’s total number is through the number of tests performed on suspected people around the country. This signifies that the number of confirmed cases vary in the country’s tests performed. Testing is the significant player in reducing the pandemic and its spread across the country. Figure 5 and Figure 6 indicate that some countries like Australia and South Korea have a positive rate of less than 1% of hundreds, or even thousands of tests to find one case in these countries. Other countries such as Mexico and Nigeria have positive rates of 20–50%. In these countries, a case is found for every few tests conducted. Countries with an extremely high positive rate are not likely to be testing widely enough to find all cases [47]. In the USA, the total tests performed across all states was 52,942,145 with reported positive tests of 5,046,506 and a positive rate of 10%, while in Saudi Arabia, the total tests performed was around 3,289,692, around an 8–10% positive rate. The WHO has recommended a positive rate of just about 3–12% as a common point of reference for sufficient testing [48]. While the situation is different in other countries such as Egypt, the total tests performed was about 135,000 and the positive cumulative cases recorded about 92,000 infected cases with 66.6% of the total tested numbers. These results prove the significance of COVID-19 pandemic tests and its role during pandemic management, especially given that these testing procedures are pushing healthcare systems around the world into serious stress. As such, there is a massive challenge for test kits and the rate of their production. In the United States, diagnostic kits manufactured by the Centers for Disease Control and Prevention caused the early scatter of the disease to be undetected for weeks.

There is strong evidence between the country decision to do more screening tests across the country and the total number of deaths and infected cases. The above figure shows the significance of testing against the pandemic and its rate of spread. Without accurate information about who is infected by the virus, we have no way to understand the pandemic. We cannot figure out which countries are doing well, and which countries are only reporting cases and deaths. Moreover, we need screening tests to understand the data on confirmed cases as it is important to know how much testing for COVID-19 the country has performed. This study confirms that the ultimate response for community health is to start testing as early as possible, which will lead directly for the rapid detection of cases, fast treatment for infected people, and instant isolation for those who are suspected in order to avoid the spread of disease.

### 3.2. Suggested COVID-19 Prevention Measures Strategy for the Saudi Arabian Community

**Figure 7:**
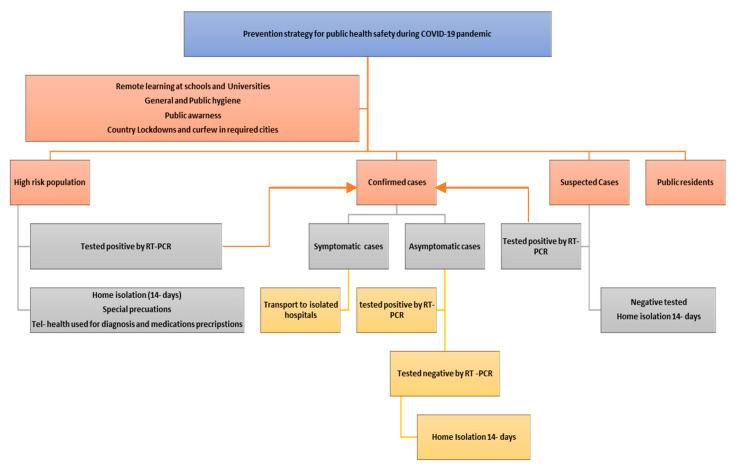
A suggested COVID-19 preventive measures strategy that is valid to the circumstances in Saudi Arabia. *Rt-PCR is defined as reverse transcription polymerase chain reaction laboratory test.*

The USA uses an instinctive model that adopts the latest techniques of a standard infectious disease model to create COVID-19 infections and death predictions for the U.S. across all 50 U.S. states. These predictions include 6.4 billion people and account for >97% of all global COVID-19 deaths. Infection assessment involves all infected people with the SARS-CoV-2 virus, not just those that had a COVID-19 test and tested positive. As of July, the model estimation of the true number of infected people in the U.S. is around 5–8 times higher than the reported cases. The current total death is 154,444, while the total projected number is 228,800 deaths by 1 Nov 2020 (Range: 198–271 k). Now, the infected rate is 2.0% (1 in 50) whereas the total infected is 12.0% (1 in 8) [49]. However, the predictions for other countries shows that in Saudi Arabia, the current death mean is 2887 while the projected number is 3700 up to 1 November 2020, with additional deaths at 74% with the 97.5th percentile of 8165 and 2.5th percentile death projected of 3514. In China, the total current death number is 4667, while the projected number is 4818 in conjunction with the additional death mean of 3% [50]. These results show the significance of performing preventive measures in reducing infection and death rates around different countries that have different socioeconomic status. Preventive measures through pandemics include self-imposed measures such as social distancing, wearing masks in public spaces, self-hygiene, and remote working and learning. This study provides a clear and achievable strategy for the prevention, early detection, and control of COVID-19 as shown in Figure 7. Once this pandemic ends, one will be capable of evaluating the health, social, and economic effects of this global disaster and will be competent to understand the lessons, particularly in terms of public and global health for any future similar pandemics [51].

## 4. Conclusions

As we are looking forward to the development of antiviral medications and a COVID-19 vaccine, public health measures are the safest means to struggle against the Covid-19 pandemic. Our study proves that the most definitive rejoinder for public health is performing screening tests as early as possible to facilitate the rapid detection of infected cases, fast treatment, and instant isolation for suspected and high risk cases. In Saudi Arabia, it is noticeable that most of the preventive measures taken by the government were implemented by March 15, reflecting only 300 cases out a population of 35 million of about 0.03% per 1 million. Our study shows that preventive measures through pandemics including self-imposed measures such as social distancing, wearing masks in public spaces, self-hygiene, and remote working and learning may have a great effect on the spread of the pandemic spread. Moreover, our study revealed the significance of performing preventive measures in reducing the infection and death rates around Saudi Arabia by 27%, while in other countries, it reduced the death rate from 10 to 73%. This study provides a clear and achievable strategy for the prevention, early detection, and control of COVID-19 spread.
